# Detection of the Prognostic Gene CYB5D2 in Cervical Squamous Epithelial Lesions

**DOI:** 10.1155/mi/2360364

**Published:** 2025-05-13

**Authors:** Yanan Liu, Guoqiang Zhao, Yanqing Kong, Fengyuan Zhou, Tong Zhang, Xiaohang Chen, Haiyan Hu, Fengxiang Wei

**Affiliations:** ^1^Genetic Laboratory, Longgang District Maternity and Child Healthcare Hospital of Shenzhen City (Longgang Maternity and Child Institute of Shantou University Medical College), Shenzhen 518000, China; ^2^Binzhou People's Hospital Blood Transfusion Department, Binzhou 256600, China; ^3^Shenzhen Maternity and Child Healthcare Hospital, Shenzhen 518028, China; ^4^Gynecology Department, Longgang District Maternity and Child Healthcare Hospital of Shenzhen City, Shenzhen 518000, China

**Keywords:** cervical cancer, CYB5D2, precancerous lesions, prognosis, screening

## Abstract

CYB5D2 is a novel tumor suppressor gene that exhibits ectopic expression in various tumors. This study explored its significance in cervical cancer screening and prognosis by examining its expression in cervical precancerous lesions and cancer tissues and analyzing follow-up data. CYB5D2 expression was comprehensively assessed in 112 clinical samples, combined with routine cervical cancer screening methods to evaluate its early detection potential. Postoperative survival data from cervical cancer patients were analyzed using Kaplan–Meier curves to examine the association between CYB5D2 protein expression and clinicopathological characteristics, as well as its prognostic implications. Results revealed a progressive downregulation of CYB5D2 expression with advancing cervical lesions. Immunohistochemical detection of CYB5D2 protein outperformed ThinPrep cytology test (TCT), DNA aneuploidy analysis, and HR-HPV E6/E7 mRNA testing (mRNA expression of the E6 and E7 genes in high-risk HPV virus) in diagnosing high-grade squamous intraepithelial lesions (HSIL+) of the cervix. Combined testing of TCT, HR-HPV E6/E7 mRNA, and CYB5D2 achieved 100% sensitivity and negative predictive value for HSIL+. In conclusion, low CYB5D2 expression was identified as an independent risk factor for progression-free survival (PFS) in cervical cancer patients. Incorporating CYB5D2 testing into routine screening protocols for squamous cell lesions, along with TCT and HPV testing, may enhance diagnostic efficiency and provide prognostic value for adverse outcomes.

## 1. Introduction

Cervical cancer incidence has declined in developed nations due to screening and vaccination, yet it remains a major burden in low- and middle-income areas, which see 85% of global cases and deaths [1, 2]. Lingering high-risk human papillomavirus (HR-HPV) infection is a key driver of cervical intraepithelial neoplasia (CIN) and cancer progression [3]. However, HPV infection alone isn't enough to cause cervical cancer [4]. Methods like HR-HPV E6/E7 mRNA analysis and DNA ploidy quantitative analysis have gradually gained widespread usage for early screening of cervical precancerous lesions [5]. Interestingly, some cervical cancer patients are HPV-negative, highlighting the need to identify complementary molecular markers. Recent studies implicate CYB5D2 (cytochrome B5 domain-containing protein 2), a tumor suppressor localized on chromosome 17p13, plays a role in cervical cancer development [6, 7]. This heme-binding protein regulates cell proliferation and differentiation, with decreased expression tied to cervical cancer progression [8]. However, its diagnostic and prognostic utility across cervical lesion stages remains unexplored. This study evaluates CYB5D2 expression in cervical lesions using droplet digital polymerase chain reaction (ddPCR) and immunohistochemistry and correlates findings with TCT, HR-HPV E6/E7 mRNA, and DNA ploidy data to assess its clinical significance. Furthermore, by using tissue microarrays and survival data, we analyze how CYB5D2 relates to clinicopathological traits and prognosis. This research seeks to uncover the diagnostic potential and prognostic value of CYB5D2 in cervical cancer and look into its future clinical applications.

## 2. Materials and Methods

### 2.1. Clinical Specimens

From September 2022 to June 2023, 112 cases of cervical lesions were collected. histopathological examination confirmed 30 cases of chronic cervicitis, 20 cases of low-grade squamous intraepithelial lesions (LSILs), 38 cases of high-grade squamous intraepithelial lesions (HSILs), and 24 cases of squamous cell carcinoma (SCC). Cervical tissues were analyzed using immunohistochemistry, HR-HPV E6/E7 mRNA testing, and ddPCR detection. Cervical exfoliated cells (both tissue and exfoliated cells from the same individual) were employed for TCT and DNA ploidy testing. Details of all samples are shown in Table 1. The study was approved by the Ethics Committee, written informed consent was obtained from all registered patients, and all relevant investigations were conducted in accordance with the principles of the Declaration of Helsinki. The inclusion criteria are as follows: (1) women aged 25–64 years; (2) no history of cervical cancer or hysterectomy; (3) completion of TCT and DNA ploid analysis; (4) no pregnancy or pregnancy termination within 8 weeks; and (5) understand the research procedures and participate voluntarily. Two tissue microarrays were utilized: CIN102 (10 normal, 82 CIN samples) purchased from Wuhan Funiu Biotechnology Co., Ltd. and CEC1601 (80 cervical cancer tissues) purchased from Shanghai Eichi Biotechnology Co., Ltd. The chip sample included patients aged 26–87 years. Complete follow-up data were available for all 80 cancer patients, with 1-year and 5-year survival rates of 99% and 90%, respectively.

### 2.2. Histopathological Examination

Two pathologists made histopathological diagnoses according to WHO criteria, categorizing samples into normal squamous cells with or without inflammation (normal or cervicitis), LSIL, HSIL, and SCC. Histological and pathological examination results were utilized as the reference standard, categorizing all diagnoses into two groups: the negative group, encompassing normal cervix and LSIL (including LSIL and chronic cervical inflammation), referred to as the LSIL- group; and the positive group, comprising HSIL and more severe disease (including HSIL and SCC), termed as the HSIL+ group.

### 2.3. ThinPrep Cytology Test Detection

Exfoliated cervical cells were obtained from the transition zone between squamous and columnar epithelium at the outer margin of the cervix. They were collected and preserved in ThinPrep Cytology Test (TCT) preservation solution by a trained gynecologist. ThinPrep specimens were prepared according to the manufacturer's instructions (Holloje, China). The cytological slides were diagnosed by two pathologists, and the TCT results were read according to the classification criteria recommended by The Bethesda System (TBS) in 2001. In this study, when the TCT results were equal to and greater than Atypical Squamous Cells of Undetermined Significance (ASCUS),they were judged as the positive group, while No Intraepithelial or Malignant Lesions (NILM) was judged as negative group.

### 2.4. HR-HPV E6/E7 mRNA Detection

HPV E6/E7 mRNA was detected in HPV-positive patients following the manufacturer's instructions precisely (XinxiangKotia, China). Both the viral mRNA and the preamplified probe hybridized with the solid-coated probe. When the amplified probe was bound to the preamplified probe, a complex was formed. The luminescent probe labeled with alkaline phosphatase was hybridized with the solid phase complex, and subsequently, the luminescence value of the sample was detected using a QuantiVirus luminescence analyzer. Results were calculated by Diacarta software, recorded as relative light units, and converted to copy number. HR-HPV E6/E7 mRNA copy number of 1 or more was considered positive.

### 2.5. DNA Ploidy Quantitative Analysis and Detection

The exfoliated cervical cells were stained with Feulgen (Abcam, UK). The specimens were first scanned by automatic quantitative imaging cell technology with at least 6000 cells scanned per smear. Then, the samples were analyzed by MotiCytometer (Motic, China). Histograms and cell dot plots of DNA ploidy analysis were generated. Interpretation of DNA ploidy quantitative analysis results: cells with a DNA Index (DI) > 2.5 were considered aneuploid. The suspicious result involved the identification of a limited number of 1–2 aneuploid cells. Abnormalities were defined when 3–10 or more aneuploid cells were detected, and colposcopic biopsy was recommended. In this study, the absence of aneuploid cells and the presence of only a small number (1–2 aneuploid cells) were classified as negative results, while the detection of 3–10 or more aneuploid cells was categorized as positive results.

### 2.6. Immunohistochemical Detection

The paraffin sections were prepared and subjected to antigen retrieval using a microwave with citrate buffer. The tissue sections were incubated overnight at 4°C with antibodies against CYB5D2 (1:100, Santa, USA). A sheep anti-IgG antibody labeled with HRP (1:25, Seville, China) was then applied, followed by staining with diaminobenzidine. The stained sections were dehydrated and sealed. Normal cervical tissue samples served as positive controls, while PBS was used instead of the CYB5D2 antibody for negative controls. A pathological section scanner (NanozoomerS210, Japan) was used for scanning, and immunohistochemical scores were independently evaluated by two pathologists in a blind manner. The CYB5D2 protein was mainly located in the membrane of the cervical tissue and partly in the cytoplasm. The criteria were as follows: the staining intensity was divided into 0 (no staining), 1 (weak staining), 2 (moderate staining), and 3 (strong staining). The estimated percentage of each category (from 0% to 100%) was as follows: 0 (< 5%), 1 (5%–25%), 2 (26%–50%), 3 (> 50%). A semiquantitative histological score was calculated by multiplying the two results. In this study, a score of 3 or more was classified as positive immunohistochemical reaction expression, and a score less than 3 was classified as negative expression.

### 2.7. Cervical Microarray Tissue by Immunofluorescence Detection

Cervical microarray tissue samples were subjected to antigen repair using repair buffers, followed by sealing with goat serum and overnight incubation at 4°C with the primary antibody CYB5D2 (1:55, Santa, USA). Subsequently, they were incubated with a fluorescently labeled Donkey anti-Rabbit IgG (1:150, Affinity Biosciences, China). After staining with 4',6-diamino-2-phenyllindodihydrochloric acid (DAPI), the stained and sealed samples were observed under a confocal laser microscope. Image J software was used to detect the fluorescence signal intensity as an indicator of the relative expression of the CYB5D2 protein.

### 2.8. Digital Droplet PCR Detection

Total RNA was extracted according to the manufacturer's instructions (Tiangen Biochemical, China), and cDNA was synthesized using a reverse transcription kit (Abm, Canada) following the manufacturer's instructions. Amplification was performed on a digital droplet PCR reaction system (TargetingOne, China). A microdrop digital PCR reaction mixture of 30 μL was prepared for each sample, then 30 μL of the reaction mixture and 180 μL of droplet-generating oil were added to the droplet generating chip, and the chip was placed in Drop Maker M1 (TargetingOne, China) according to the manufacturer's instructions to generate water-in-oil emulsion droplets. Target gene mRNA sequence were as follows: CYB5D2-Forward：5'-CTGTATAAGCCAGGTGCTAAGG- 3'; CYB5D2-Reverse：5'-TTTCTGTGTGGAGGGTTGTC- 3'; CYB5D2-Probe：5'-TGCGTGTGTGTGAGAACCACCG- 3'. The chip was placed in the chip reader to detect the fluorescence signal of the droplets. Poisson distribution analysis of the data was performed using dedicated software to obtain the target RNA copy number.

### 2.9. Statistical Analysis

SPSS 20.0 software was utilized for conducting statistical analysis. Continuous variables were compared between groups using the Student's *t*-test, while categorical variables were assessed using the Chi-square test. The mean ± standard deviation (SD) was used to express data obtained from a minimum of three independent experiments. The sensitivity, specificity, Youden index, positive predictive value, and negative predictive value were calculated through a comparison of the area under the Receiver Operating Characteristic (ROC) curve (AUC) using the Mann-Whitney U test. Survival curves were constructed using Kaplan–Meier method, and univariate analysis of cervical cancer was performed using log-rank test. Multivariate analysis was conducted utilizing COX regression analysis. A significance level of *p*  < 0.05 was considered statistically significant in all analyses undertaken.

## 3. Results

### 3.1. Expression of CYB5D2 in Cervical Cancer and Precancerous Lesions of Different Grades

**Figure 1:**
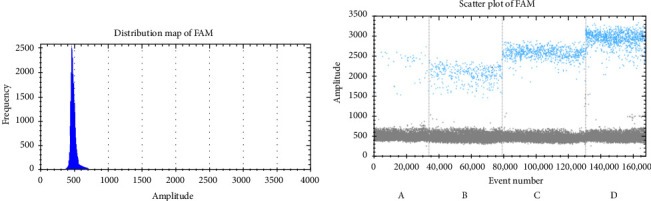
CYB5D2 detection results: Black and gray microdroplets were no amplification negative microdroplets, blue microdroplets were PCR positive amplification microdroplets in FAM channel (A) cervical squamous cell carcinoma, (B) HSIL, (C) LSIL, (D) chronic cervicitis).

**Figure 2:**
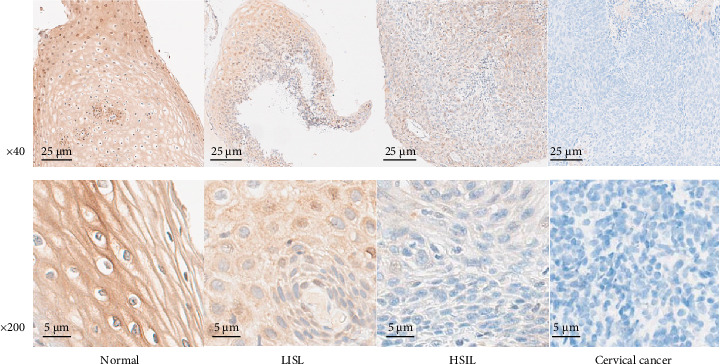
CYB5D2 expression at different levels of cervical lesions under immunohistochemical staining microscope (40×, 200×).

The ddPCR results showed that the copy numbers of CYB5D2 in chronic cervicitis, LSIL, HSIL, and cervical SCC were 908.43 ± 85.71, 689.50 ± 52.45, 298.89 ± 30.77, and 82.13 ± 10.20 copies/μL, respectively (Figure 1). Immunohistochemical analysis revealed that 39.29% (44 out of 112) of patients with cervical lesions showed positive expression of the CYB5D2 protein. Specifically, the positive expression rates for chronic cervicitis, LSIL, HSIL, and cervical SCC were 83.33% (25 cases), 65% (13 cases), 10.52% (four cases), and 8.33% (two cases) respectively (Figure 2). Evidently, a decrease in CYB5D2 expression was observed with an increase in the severity of cervical lesions.

### 3.2. Comparison of Diagnostic Efficacy of TCT, HR-HPV E6/E7 mRNA, DNA Ploidy Quantitative Analysis, Single CYB5D2 Protein Detection, and Different Combined Detection Schemes for Cervical Cancer and Precancerous Lesions

#### 3.2.1. Separate Detection Scheme

TCT results that among the 112 patients with cervical lesions, 37 (33.04%) tested negative for NILM while 75 (66.96%) tested positive, including 28 (25.00%) with ASCUS, 12 (10.71%) with LSIL, and 35 (31.25%) with ASC-H and HSIL. Histopathological findings revealed that 50 cases (44.64%) in the LSIL group, exhibited inflammation in 30 cases (26.79%) and LSIL in 20 cases (17.85%). In the HSIL+ group, there were 62 cases (55.36%), including 38 cases (33.93%) of HSIL and 24 cases (21.43%) of cervical SCC. The HR-HPV E6/E7mRNA test results indicated that out of the 112 patients with cervical lesions, 81 cases (72.32%) tested positive for HR-HPV E6/E7mRNA, with positivity rates of 10 cases (33.33%), 16 cases (80%), 33 cases (86.84%), and 22 cases (91.66%) for chronic cervicitis, LSIL, HSIL, and cervical SCC, respectively. Quantitative analysis of DNA ploidy revealed that among the 112 patients with cervical lesions, 68 cases (60.71%) exhibited positive results (≥3 aneuploid abnormalities). The percentages for chronic cervicitis, LSIL, HSIL, and cervical SCC were 5 cases (16.67%), five cases (16.67%), 28 cases (73.68%), and 24 cases (100.00%), respectively. Negative results (no and 1–2 aneuploid abnormalities) were found in 44 cases (39.29%), with percentages of 25 cases (56.82%) for chronic cervicitis, nine cases (20.45%) for LSIL, and 10 cases (22.72%) for HSIL, and 0 cases (0.00%) for cervical SCC, respectively. In the single detection scheme, the sensitivity (90.32%), specificity (76.00%), positive predictive value (82.35%), negative predictive value (86.36%), and Yoden index (0.66) of CYB5D2 protein detection for the diagnosis of cervical HSIL+ were higher than those of any of the other three detection methods (Table 2).

#### 3.2.2. Comprehensive Testing Scheme

The joint test scheme determines the screening positive result based on the positive outcome of any index in the joint test, while the absence of positive results in all indicators was used to determine a negative screening result. The combined detection of TCT and CYB5D2 showed 60 positive cases and two negative cases in the HSIL+ group, as well as 23 positive cases and 27 negative cases in the LSIL- group. The sensitivity (96.77%) and negative predictive value (93.10%) of TCT and CYB5D2 detection in the diagnosis of cervical HSIL+ surpass those of any other combined detection methods. DNA ploidy quantitative analysis combined with CYB5D2 detection identified 59 positive cases and three negative cases in the HSIL+ group, as well as 16 positive cases and 34 negative cases in the LSIL- group. The specificity (68.00%), positive predictive value (78.67%) and Yoden index (0.63) for cervical HSIL+ diagnosis were found to be higher than those of any other two combined detection methods. The results of CYB5D2 combined with HR-HPV E6/E7 mRNA and DNA ploidy quantitative analysis showed that 62 cases were positive and 0 case was negative in HSIL+ group. In LSIL- group, 26 cases were positive and 24 cases were negative. The sensitivity and negative predictive value of CYB5D2 combined with HR-HPV E6/E7 mRNA and DNA ploidy were the highest (100.00%), which was the same as that of CYB5D2 combined with HR-HPV E6/E7 mRNA and TCT. The specificity (48.00%), positive predictive value (70.45%) and Youden index (0.48) were higher than those of any of the other three combined detection methods (Table 2).

#### 3.2.3. Receiver Operating Characteristic Curve Analysis of Various Detection Methods for Diagnosing HSIL+

**Figure 3:**
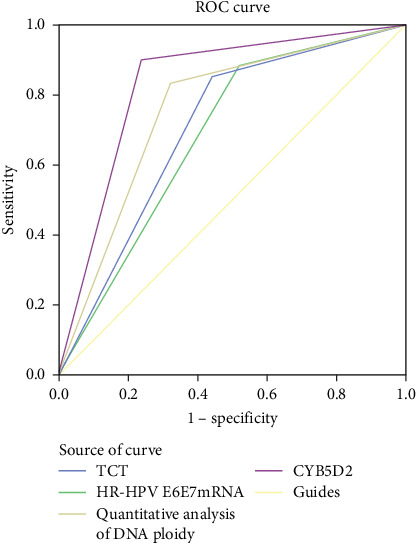
ROC curve of HSIL+ induced by TCT, HR-HPV E6/E7 mRNA, DNA ploidy quantitative analysis, and CYB5D2 protein detection.

The ROC curve results of TCT, HR-HPV E6/E7 mRNA, DNA ploidy quantitative analysis, and CYB5D2 protein detection for HSIL+ were presented in Figure 3 and Table 3. Among these tests, the diagnostic value for HSIL+ ranked as follows: CYB5D2 protein detection (AUC = 0.832) > DNA ploidy quantitative analysis (AUC = 0.759) > TCT (AUC = 0.707) > HR-HPV E6/E7 mRNA (AUC = 0.684). Collectively, these data demonstrate that CYB5D2 detection exhibits a high level of diagnostic accuracy for identifying HSIL+.

### 3.3. Relationship Between CYB5D2 Expression and Clinicopathological Features of Cervical Cancer

**Figure 4:**
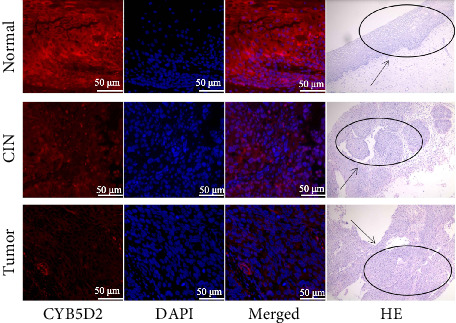
Fluorescence expression of CYB5D2 in different cervical lesions (CIN: cervical intraepithelial neoplasia).

The relative fluorescence expression of CYB5D2 protein in normal cervical tissues, CIN tissues, and cervical cancer tissues was 91.38 ± 7.45, 58.06 ± 3.82, and 16.04 ± 3.60, respectively (Figure 4). With the increasing degree of cervical lesions, the relative expression of CYB5D2 protein fluorescence gradually decreased, showing a downward trend. In this study, 80 cases of cervical cancer were categorized into a low CYB5D2 expression group and a high CYB5D2 expression group based on the median relative expression level of CYB5D2 protein fluorescence. Significant differences were observed in the expression of CYB5D2 among cervical cancer patients with varying FIGO stages, tumor size, myometrial infiltration, and differentiation degree. However, no significant difference was found in relation to age, lymph node metastasis or HPV16 infection (Table 4).

### 3.4. Factors That Influence Progression-Free Survival After Surgical Treatment for Cervical Cancer

**Figure 5:**
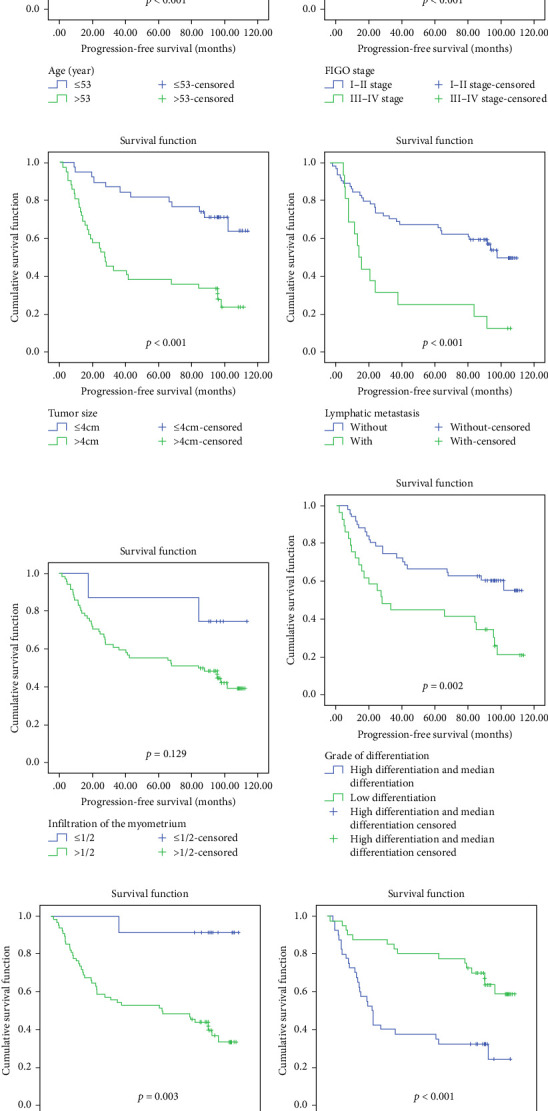
(A) Analysis of PFS curves in patients aged ≥53 years and patients aged < 53 years; (B) PFS curve analysis of FIGO stage Ⅰ–Ⅱ and Ⅲ–Ⅳ patients; (C) PFS curve analysis of patients with tumor size ≤4 cm and patients with tumor size > 4 cm; (D) PFS curve analysis of patients without lymph node metastasis and patients with lymph node metastasis; (E) PFS curve analysis of patients with ≤1/2 and >1/2 myometrium infiltration; (F) PFS curve analysis of highly differentiated + moderately differentiated patients and poorly differentiated patients; (G) PFS curve analysis of HPV16 patients without infection and patients with infection; (H) PFS curve analysis of patients with low CYB5D2 expression and patients with high CYB5D2 expression.

In a cohort of 80 cervical cancer patients with complete follow-up data, recurrence was frequently observed in the urinary system, pelvic wall, and parafervix, while metastasis most commonly occurred in the abdominal cavity, lung, bladder, and colon. The mean progression-free survival (PFS) was 70 months. Kaplan–Meier curve analysis was utilized to assess the impact of CYB5D2 expression and other pathological features on PFS in cervical cancer patients. The findings revealed that PFS for the low CYB5D2 expression group was 49 months, whereas PFS for the high CYB5D2 expression group was 90 months. As cervical cancer progressed, there was a decrease in CYB5D2 expression which exhibited a negative correlation with patient PFS (Figure 5). The clinicopathological characteristics associated with influencing factors for postoperative PFS include age ≥ 53 years, FIGO stage III–IV, tumor size larger than 4 cm, lymph node metastasis, low tumor differentiation, and HPV16 infection.

After excluding data from other patients with incomplete information, univariate Log-rank and multivariate COX regression analyses were conducted to determine whether CYB5D2 served as an independent predictor of PFS in cervical cancer patients. The results of the univariate Log-rank analysis indicated that age ≥53 years old, FIGO stage III–IV, tumor size larger than 4 cm, lymph node metastasis, low tumor differentiation, HPV16 infection, and low CYB5D2 expression were all identified as risk factors for postoperative PFS. Furthermore, the multivariate COX regression analysis revealed that FIGO stage III–IV confirmed by pathology during operation, presence of lymph node metastasis, and HPV16 infection were associated with poor prognosis along with low CYB5D2 expression (Table 5).

## 4. Discussion

Timely screening and treatment in the precancer stage is an effective way to prevent cervical cancer [9]. Currently, the standard cervical disease screening protocol in most hospitals involves TCT combined with HPV testing, and some hospitals also recommend DNA ploidy quantitative analysis to enhance diagnostic accuracy for cervical lesions [10, 11]. This study evaluated the newly identified tumor suppressor CYB5D2, comparing its diagnostic efficacy for cervical HSIL+ with TCT, HR-HPV E6/E7 mRNA, and DNA ploidy quantitative analysis—both alone and in combined detection schemes. Our results showed that CYB5D2 protein detection achieved higher sensitivity (90.32%), specificity (68.00%), positive predictive value (82.35%), negative predictive value (77.27%), and Youden index (0.66) for HSIL+ diagnosis than the other three single methods. This indicates CYB5D2 is a more reliable and accurate biomarker for HSIL+ compared to TCT, HR-HPV E6/E7 mRNA, and DNA ploidy analysis. Notably, the combination of TCT, HR-HPV E6/E7 mRNA, and CYB5D2 achieved 100% sensitivity and negative predictive value for HSIL+ lesions, effectively reducing missed diagnoses. With an AUC area of 0.832, CYB5D2 exhibits superior diagnostic efficiency for cervical precancerous lesions. Our ddPCR and immunohistochemistry data revealed that a progressive decrease in CYB5D2 expression with lesion severity, with 91.67% of cervical cancer tissues showing downregulation compared to chronic cervicitis. This aligns with prior reports that CYB5D2 as a tumor suppressor was detected in the gene spectrum of acute lymphoblastic leukemia [12]. In the study of Xie et al. [6], it was found that CYB5D2 expression was reduced in 87.5% (35/40) of SCCs compared with normal cervical tissues. These findings suggest that CYB5D2 plays a critical role in inhibiting malignant progression and could serve as a biomarker for cervical lesion deterioration. According to the fluorescence expression of CYB5D2 protein in 80 cases of cervical cancer combined with related clinicopathological features, univariate and multivariate variable analysis showed FIGO stage III–IV, lymph node metastasis, HPV16 infection and low CYB5D2 expression as independent predictors of PFS in patients with cervical cancer after surgery. At the same time, Kaplan–Meier curves showed that the PFS of patients with low CYB5D2 expression was significantly less than that of patients with high CYB5D2 expression, further indicating that CYB5D2 plays a role in inhibiting cervical lesions and can be used as a good indicator for evaluating the prognosis of cervical cancer. Limitations of this study include its retrospective design and small sample size, which warrant validation in larger cohorts. Thus, we recommend integrating CYB5D2 testing into traditional screening protocols to improve biological assessment and minimize unnecessary colposcopies and biopsies. However, cost–benefit analyses are needed before widespread implementation.

To comprehensively elucidate the role of CYB5D2 in cervical cancer, we conducted a thorough analysis of data from the Gene Expression Omnibus (GEO) database. While intratumoral heterogeneity in gene expression profiles has been documented in cervical cancer patients, CYB5D2 expression did not exhibit such heterogeneity [13]. In early-stage cervical cancer, pelvic lymph node metastasis is a major prognostic factor. However, analysis of 20 lymph node-negative (N0) and 19 lymph node-positive (N+) patients revealed no significant differences in CYB5D2 expression [14]. In another study, CYB5D2 expression was found to decrease from carcinoma in situ (CIS) to SCC, suggesting an inverse correlation between CYB5D2 expression and tumor malignancy. However, the small sample size limits the statistical robustness of this conclusion [15]. We further explored the correlation between CYB5D2 expression and HPV infection. CYB5D2 expression was significantly lower in primary tumor samples compared to normal cervical tissues, indicating its potential role as a tumor suppressor. These findings suggest that CYB5D2 may serve as a diagnostic marker for early tumor detection, consistent with our results [16]. Additionally, we analyzed CYB5D2 expression across different stages of cervical cancer. CYB5D2 expression was relatively high in stage 1B tumors but significantly decreased by stage 4A [17]. This pattern suggests that CYB5D2 may actively inhibit tumor progression in the early stages by suppressing the abnormal proliferation of tumor cells through multiple pathways. As the tumor progresses, tumor cells may develop mechanisms to suppress CYB5D2 expression or inactivate its function. Thus, changes in CYB5D2 expression could serve as a more precise biomarker for the diagnosis and staging of cervical cancer. Given the tumor-suppressive role of CYB5D2, its expression level is likely closely associated with patient prognosis. Analysis of CYB5D2 expression before and after immunotherapy revealed statistically significant differences, indicating that CYB5D2 has the potential to serve as a biomarker for predicting the efficacy of immunotherapy [18].

Extensive literature reviews indicate CYB5D2 exerts tumor-suppressive effects across multiple malignancies, including breast cancer, hepatocellular carcinoma, lung cancer, and clear cell renal cell carcinoma (ccRCC). Its downregulation correlates with tumor progression, paralleling its role in cervical cancer and suggesting a universal regulatory mechanism in cancer development. In breast cancer, CYB5D2 regulates mitosis, DNA metabolism, and repair processes to inhibit tumorigenesis [19]. In hepatocellular carcinoma, low CYB5D2 expression negatively correlates with TGF-*β*, and overexpression partially counteracts TGF-*β*-mediated cell proliferation and migration [20]. In ccRCC, CYB5D2 serves as a prognostic marker associated with early-stage prediction, with downregulation driving disease progression [21]. Similarly, in cervical cancer, CYB5D2 inhibits epithelial–mesenchymal transition (EMT) by upregulating E-cadherin and downregulating N-cadherin, Snail, and Twist, thereby reducing the invasiveness and metastatic ability of tumor cells [22]. Our GEO database analysis revealed an interesting correlation between CYB5D2 expression levels and HPV infection types in cervical cancer cell lines. Among the cell line samples examined, SiHa (HPV16-positive) and C33A (HPV-negative) displayed higher CYB5D2 expression, whereas HeLa (HPV18-positive) exhibited low expression. This suggests a potential association with HPV type and inherent cell line traits, though further validation is needed due to the limited sample size [16]. Studies demonstrate CYB5D2 downregulation in cervical cancer, which may cooperate with HPV-induced microenvironmental changes to drive malignant transformation. On the one hand, HPV E6 protein disrupts DNA repair and cell cycle regulation by degrading P53 [23]. On the other hand, CYB5D2 reduction correlates with mutations in genes such as PIK3CA and P53 [19]. Therefore, the downregulation of CYB5D2 may further exacerbate these defects, resulting in a double-hit effect. Furthermore, our analysis of the GEO database has uncovered differential expression of CYB5D2 before and after immunotherapy, suggesting its possible involvement in the immune response and its correlation with the efficacy of immunotherapy. Studies demonstrate that low-risk genes like CYB5D2 negatively correlate with immune cell infiltration, with high CYB5D2 expression associating with reduced immune infiltration [24, 25]. GEO database analysis reveals differential expression of immune checkpoint molecules (such as GZMB, IFNG, FOXP3) during the progression of cervical cancer, suggesting CYB5D2 modulates the tumor immune microenvironment by regulating the infiltration of immune cells into tumor tissues and thereby affecting the prognosis of patients. The interaction network constructed through the STRING database suggests significant interactions between CYB5D2 and multiple genes (Supporting Information Figure S1). For instance, PAQR5 expression inversely correlates with TGF-*β* signaling, and reduced PAQR5 levels are associated with aggressive clinicopathological features, including advanced stage, high grade, lymph node metastasis, and distant metastasis, in ccRCC [26]. Our findings suggest CYB5D2 may cross-talk with the TGF-*β* pathway via molecules like PAQR5, offering mechanistic insights for future studies. Structurally, CYB5D2 binds heme via its cytochrome b5 domain, with residue D86 playing a critical role. The transmembrane domain, particularly amino acid R7, mediates tumor-suppressive activity. R7P and R7G mutants retain heme-binding capacity [6]. The heme-binding activity of CYB5D2 is closely related to oxidative stress, which may affect tumor invasion and metastasis by regulating the degradation of the extracellular matrix and cell motility. CYB5D2 colocalizes with cytochrome P450 reductase (CYPOR) and knockdown reduces the activity of CYP3A4 and affects the cell's tolerance to oxidative stress [27]. In cervical cancer, oxidative stress alterations likely impact cell survival and function, though the underlying mechanisms require further investigation.

Limitations of this study and suggestions for follow-up research: Although CYB5D2 is involved in multiple pathways, its upstream and downstream targets and the regulated signaling pathways have yet to be fully elucidated. The focus of this study's experiments was on enhancing the diagnostic efficiency of traditional cervical cancer screening through CYB5D2. However, the varying expression levels of CYB5D2 in different HeLa cell lines (such as SiHa, HeLa, and CaSki) as shown by GEO database analysis require further exploration through more experiments. The differential expression of CYB5D2 before and after immunotherapy is also a novel finding. To expand the scope of our research, we plan to incorporate 3D cell culture technology, which can more accurately simulate the in vivo tumor microenvironment. Additionally, by establishing animal models, we aim to validate our findings in a physiological context and enhance the clinical relevance of the research.

Future directions include exploring CYB5D2's molecular mechanism, verifying its diagnostic and prognostic potential, developing targeted therapies based on its tumor-suppressing function, and studying its role in the immune microenvironment and interactions with other cancer-related genes and pathways.

## 5. Conclusion

This study investigated CYB5D2 expression in cervical lesions. Core findings show that CYB5D2 is differentially expressed in precancerous and cancerous cervical tissues. Its expression decreases as cervical lesions worsen. In terms of diagnosis, combined detection of TCT, HR-HPV E6/E7 mRNA, and CYB5D2 can achieve 100% sensitivity and negative predictive value for diagnosing HSIL+ lesions, reducing missed diagnoses. Regarding prognosis, low CYB5D2 expression in cervical cancer tissues is linked to enhanced growth and invasion. It is also an independent risk indicator for poor PFS in cervical cancer patients. However, large prospective studies are needed to confirm its prognostic value, and more experiments are required to clarify its biological role in cervical cancer

## Figures and Tables

**Table 1:** Cervical tissue clinical information.

Group	Number [*n* (%)]
Fresh cervical tissue	112
Number of patients analyzed	112
Female age—year (range)	25–64 (year)
Histopathological grading	—
LSIL−	—
Chronic cervicitis	30
LSIL	20
HSIL+	—
HSIL	38
Squamous carcinoma of the cervix	24
Tissue chip	172
Age (year)	—
＜50	75 (43.60)
≥50	97 (56.40)
Type of specimen	—
Normal cervical tissue	10 (5.81)
Cervical intraepithelial neoplasia tissue	82 (47.67)
Cervical cancer tissue	80 (46.51)
Types of cervical cancer	—
squamous-cell carcinoma	80 (100.00)
Differentiation degree	—
High differentiation	14 (17.50)
Median differentiation	37 (46.25)
Low differentiation	29 (36.25)
FIGO stage	—
Ⅰ	18 (22.50)
Ⅱ	20 (25.00)
Ⅲ	21 (26.25)
Ⅳ	21 (26.25)
Tumor size (cm)	—
≤4	38 (47.50)
＞4	42 (52.50)
Lymphatic metastasis	—
With	16 (20.00)
Without	64 (80.00)
Infiltration of the myometrium	—
≤1/2	8 (10.00)
＞1/2	72 (90.00)

**Table 2:** Diagnostic efficacy of TCT, HR-HPV E6/E7 mRNA, DNA ploidy analysis, CYB5D2 protein detection alone, and different combined detection methods for cervical lesions [*n* (%)].

			Histopathological grading			
			LSIL-		HSIL+	
			Chronic cervicitis	LSIL	HSIL	Squamous carcinoma of the cervix
DdPCR	Number of cases (*n*)		30	20	38	24
	CYB5D2 (copies/μL)		908.43 ± 85.71	689.50 ± 52.45^a^	298.89 ± 30.77^ab^	82.13±10.20^abc^

Immunohistochemistry	CYB5D2 positive		25 (83.33)	13 (65.00)	4 (10.52)	2 (8.33)
	CYB5D2 negative		5 (16.67)	7 (35.00)	34 (89.48)	22 (91.67)

TCT	NILM		22 (73.33)	6 (30.00)	7 (18.42)	2 (8.33)
	ASCUS		6 (20.00)	10 (33.33)	9 (23.68)	3 (12.50)
	LSIL		2 (6.67)	4 (20.00)	3 (7.89)	3 (12.50)
	ASC-H+HSIL		0	0	19 (50.00)	16 (66.67)
	Total (*n*)		30	20	38	24

Detection of HR-HPV E6/E7 mRNA	Number of cases (*n*)		30	20	38	24
	Positive		10 (33.33)	16 (80.00)	33 (86.84)	22 (91.66)
	Copies (X ± S)		415.15 ± 47.45	3473.56 ± 382.85^a^	6374.05 ± 782.20^ab^	9802.13 ± 1071.31^abc^
	χ^2^				18.633	
	*p* value				＜0.001	

DNA ploidy quantitative analysis	Number of cases (*n*)		30	20	38	24
	Positive	Abnormal numbers ≥3	5 (16.67)	5 (16.67)	28 (73.68)	24 (100.00)
	Negative	No obvious abnormality	18 (60.00)	5 (25.00)	4 (10.53)	0 (0.00)
		Abnormal numbers: 1–2	7 (23.33)	4 (20.00)	6 (15.79)	0 (0.00)
		χ^2^			31.222	
		*p* value			<0.001	

			Number of positive cases (*n*)		χ^2^	*p* Value
			LSIL-	HSIL+		
			Chronic cervicitis + LSIL	HSIL + cervical squamous cell carcinoma		
Different combined detection protocols	TCT+HR-HPV E6/E7 mRNA		26	57	23.005	<0.001
	TCT+DNA ploidy		22	59	36.192	<0.001
	TCT+CYB5D2		23	60	37.187	<0.001
	HR-HPV E6/E7 mRNA+DNA ploidy		27	58	23.662	<0.001
	HR-HPV E6/E7 mRNA+CYB5D2		27	57	21.244	<0.001
	Quantitative analysis of DNA ploidy +CYB5D2		16	59	49.914	<0.001
	TCT+HR-HPV E6/E7mRNA+DNA ploidy		26	61	34.349	<0.001
	TCT+HR-HPV E6/E7 mRNA+CYB5D2		27	62	35.89	<0.001
	HR-HPV E6/E7 mRNA+DNA ploidy		26	62	37.876	<0.001
	CYB5D2					

Examination method		Sensitivity	Specificity	Positive predictive value	Negative predictive value	Youden index

TCT		85.48	56	70.67	75.68	0.41
HR-HPV E6/E7 mRNA		88.71	48	67.9	77.42	0.37
DNA ploidy		83.87	68	76.47	77.27	0.52
CYB5D2		90.32	76	82.35	86.36	0.66
TCT+HR-HPV E6/E7 mRNA		91.94	48	68.67	82.76	0.4
TCT+DNA ploidy		95.16	56	72.84	90.3	0.51
TCT+CYB5D2		96.77	54	72.29	93.1	0.51
HR-HPV E6/E7 mRNA+DNAploidy		93.54	46	68.24	85.19	0.4
HR-HPV E6/E7 mRNA+CYB5D2		91.94	46	67.86	82.14	0.38
DNA ploidy+CYB5D2		95.16	68	78.67	91.89	0.63
TCT+HR-HPV E6/E7 mRNA+DNA ploidy		98.39	48	70.11	96	0.46
TCT+HR-HPV E6/E7 mRNA+CYB5D2		100	46	69.66	100	0.46
HR-HPV E6/E7 mRNA+DNA ploidy+CYB5D2		100	48	70.45	100	0.48

*Note*: All comparisons exhibited statistically significant differences.

^a^Significant difference compared with Chronic cervicitis (*p* < 0.0001).

^b^Significant difference compared with LSIL (*p* < 0.0001).

^c^Significant difference compared with HSIL (*p* < 0.0001).

**Table 3:** Analysis of the ROC curve results of HSIL+ induced by TCT, HR-HPV E6/E7 mRNA, DNA ploidy, and CYB5D2 protein detection.

Test outcome variables	AUC	Standard error	*p* Value	95% CI	
				Lower	Upper
TCT	0.707	0.051	<0.001	0.608	0.807
HR-HPV E6/E7 mRNA	0.684	0.052	0.001	0.581	0.786
DNA ploidy	0.759	0.048	<0.001	0.666	0.853
CYB5D2	0.832	0.042	<0.001	0.749	0.914

**Table 4:** Analysis of the relationship between CYB5D2 expression and clinicopathologic features in 80 cases of cervical cancer.

Clinicopathologic feature	*n*	CYB5D2			
		Low expression	High expression	χ^2^	*p* Value
Age (year)					
<53	45	21	24	0.457	0.652
≥53	35	19	16		
FIGO stage					
I–II	38	10	28	16.241	0.001
III–IV	42	30	12		
Tumor size (cm)					
≤4	38	14	24	5.013	0.043
>4	42	26	16		
Lymphatic metastasis					
With	16	9	7	0.313	0.576
Without	64	31	33		
Infiltration of the myometrium					
≤1/2	8	1	7	5	0.025
>1/2	72	39	33		
Differentiation degree					
High differentiation + median differentiation	51	21	30	4.381	0.036
Low differentiation	29	19	10		
HPV16					
With	68	35	33	0.392	0.531
Without	12	5	7		

**Table 5:** Univariate analysis of progression-free survival after cervical cancer surgery, COX regression analysis of Log-rank, and multiple factor variables.

Influencing parameter	Univariate COX regression analysis		Multivariate COX regression analysis		
	χ^2^	*p*	HR	95% CI	*p*
Age (year)					
<53 vs ≥53	14.814	<0.001	1.6	0.722–3.549	0.247
FIGO stage					
I–II vs III–IV	47.578	<0.001	4.727	1.772–12.607	0.002
Tumor size (cm)					
≤4 vs >4	16.702	<0.001	1.061	0.500–2.251	0.878
Lymphatic metastasis					
Without versus with	12.709	<0.001	2.469	1.209–5.043	0.013
Infiltration of the myometrium					
≤1/2 vs >1/2	2.302	0.129			
Differentiation degree					
High differentiation + median differentiation versus low differentiation	9.868	0.002	0.815	0.401–1.658	0.573
HPV16					
Without versus with	8.947	0.003	12.721	1.589–101.812	0.017
CYB5D2					
Low expression versus high expression	13.313	<0.001	0.368	0.175–0.774	0.008

## Data Availability

Data sharing is not applicable to this article as no datasets were generated or analyzed during the current study.

## Supporting Information

Supporting Information Figure S1: Protein-protein interaction network of CYB5D2 generated using the STRING database (version 11.5, https://string-db.org/). Nodes represent proteins, and edges indicate interactions. The network includes key interactors such as CYB5A, CYB5B, PAQR5, PAQR6, and ANAPC16, and several others that are implicated in various cellular functions.
